# Perinatal asphyxia results in altered expression of the hippocampal acylethanolamide/endocannabinoid signaling system associated to memory impairments in postweaned rats

**DOI:** 10.3389/fnana.2015.00141

**Published:** 2015-11-03

**Authors:** Eduardo Blanco, Pablo Galeano, Mariana I. Holubiec, Juan I. Romero, Tamara Logica, Patricia Rivera, Francisco J. Pavón, Juan Suarez, Francisco Capani, Fernando Rodríguez de Fonseca

**Affiliations:** ^1^Unidad de Gestión Clínica de Salud Mental, Laboratorio de Medicina Regenerativa, Instituto de Investigación Biomédica de Málaga (IBIMA), Hospital Universitario Regional de Málaga, Universidad de MálagaMálaga, Spain; ^2^Departament de Pedagogia i Psicologia, Facultat d’Educació, Psicologia i Treball Social, Universitat de LleidaLleida, Spain; ^3^Instituto de Investigaciones Bioquímicas de Buenos Aires – Consejo Nacional de Investigaciones Científicas y Técnicas, Fundación Instituto LeloirBuenos Aires, Argentina; ^4^Facultad de Medicina, Instituto de Investigaciones Cardiológicas “Prof. Dr. Alberto C. Taquini”, Consejo Nacional de Investigaciones Científicas y Técnicas, Universidad de Buenos AiresBuenos Aires, Argentina

**Keywords:** perinatal asphyxia, hippocampus, memory, DAGLα, NAPE-PLD, CB1, PPARα, FAAH

## Abstract

Perinatal asphyxia (PA) is an obstetric complication that strongly affects the CNS. The endocannabinoid system (ECS) is a lipid transmitter system involved in several physiological processes including synaptic plasticity, neurogenesis, memory, and mood. Endocannabinoids, and other acylethanolamides (AEs) without endocannabinoid activity, have recently received growing attention due to their potential neuroprotective functions in neurological disorders, including cerebral ischemia. In the present study, we aimed to analyze the changes produced by PA in the major metabolic enzymes and receptors of the ECS/AEs in the hippocampus using a rodent model of PA. To induce PA, we removed uterine horns from ready-to-deliver rats and immersed them into a water bath during 19 min. Animals delivered spontaneously or by cesarean section were employed as controls. At 1 month of age, cognitive functions were assessed and immunohistochemical procedures were carried out to determine the expression of NeuN and glial fibrillary acidic protein, enzymes responsible for synthesis (DAGLα and NAPE-PLD) and degradation (FAAH) of ECS/AEs and their receptors (CB1 and PPARα) in the hippocampus. Postweaned asphyctic rats showed impaired recognition and spatial reference memory that were accompanied by hippocampal astrogliosis and changes in the expression of enzymes and receptors. The most remarkable findings in asphyctic rats were a decrease in the expression of NAPE-PLD and PPARα in both hippocampal areas CA1 and CA3. In addition, postweaned cesarean delivery rats showed an increase in the immunolabeling for FAAH in the hippocampal CA3 area. Since, NAPE-PLD and PPARα are proteins that participate in the biochemical process of AEs, specially the neuroprotective oleoylethanolamide, these results suggest that PA dysregulates this system. These data encourage conducting future studies using AEs as potential neuroprotective compounds in animal models of PA.

## Introduction

Perinatal asphyxia (PA) is an obstetric complication that can be triggered by different events, such as fetus lungs malfunction and alterations in the gas exchange in the placenta, leading to lack of oxygen (hypoxia) or a reduced blood flow (ischemia) to different body organs during the perinatal period (Carrera, 2006). PA affects every organ in the body and it is predominantly deleterious for the central nervous system (CNS; Kumar et al., 2007). The CNS is highly susceptible to the oxidative damage caused by PA due to its elevated concentrations of unsaturated fatty acids, high oxygen consumption, and little concentration of antioxidants (Halliwell, 1992). PA is associated with high morbimortality in term and pre-term neonates (Lawn et al., 2005). Following PA, 25% of those newborns who survive develop neurological disorders, such as cerebral palsy and several neurodevelopmental and learning disabilities (Amiel-Tison and Ellison, 1986; Mañeru et al., 2001; van Handel et al., 2007).

Several murine and rodent models are employed to study PA in experimental conditions. Two of the most renowned are the ones developed by Bjelke et al. (1991) and Vannucci et al. (1999). The former is induced in pup rats of 7 days of age by ligation of the right common carotid artery followed by exposure to an oxygen-deprived environment (Vannucci et al., 1999), while the one developed by Bjelke et al. (1991) is carried out by immersing uterus horns, containing the fetuses, into a water bath set at 37°C for different time periods. Uterus horns are obtained from pregnant rats that are ready to deliver (Bjelke et al., 1991; Capani et al., 2001). Bjelke’s model is a well-established PA model that has been extensively employed by our and other groups (Chen et al., 1995; Capani et al., 1997, 2001, 2003, 2009; Brake et al., 2000; Boksa and El-Khodor, 2003; Weitzdoerfer et al., 2004; Cebral et al., 2006; Wakuda et al., 2008; Morales et al., 2010; Saraceno et al., 2010, 2012; Strackx et al., 2010). Using this model, it has been observed that during this stage of development, PA may cause damage in several regions of the CNS, such as hippocampus, cerebellum, striatum, cerebral cortex, and substantia nigra (Bjelke et al., 1991; Capani et al., 2009; Campanille et al., 2015). The model has many advantages over others, such as: (a) the asphyxia is induced during delivery, reproducing clinical situations with more accuracy. For instance, when the circulation of the umbilical cord is altered (Capani et al., 2009); (b) it affects the whole body, mimicking global asphyxia which is the most frequent type of PA (Lubec et al., 1997; Loidl et al., 2000; Strackx et al., 2010); (c) it does not require surgical procedures which could add confounding effects; (d) both cerebral hemispheres and deep brain structures are affected by hypoxia-ischemia.

Our group has previously found that the induction of PA using the Bjelke’s model is associated to cellular and molecular changes in the hippocampus, such as focal swelling, astrogliosis, decreased phosphorylation of high and medium molecular weight neurofilaments, and synaptic alterations (Saraceno et al., 2010, 2012). At a behavioral level, we have also observed spatial reference and working memory impairments in adult and middle-aged asphyctic rats (Galeano et al., 2011, 2015).

The endocannabinoid system (ECS) is a lipid signaling system which consists of cannabinoid receptors, endocannabinoids and enzymes required to synthesize, transport and inactivate these endogenous ligands (Ueda et al., 2013). The ECS was originally identified in the CNS and it has been reported that it regulates several neurophysiological processes (e.g., embryonic cell fate in the developing brain, modulation of neural development, neurogenesis, and synaptic plasticity) primarily through the activation of the cannabinoid receptor type 1 (CB1; Alonso-Alconada et al., 2011; Serrano et al., 2012; Ueda et al., 2013; Blanco-Calvo et al., 2014). Endocannabinoids are produced on demand from membrane phospholipids and degraded by intracellular enzymes. These bioactive lipids are comprised of two classes of fatty acid derivatives structurally distinct, acylethanolamides (AEs) and monoacylglycerols (MAGs). In fact, the best characterized endocannabinoids are arachidonoylethanolamide (AEA or anandamide) and 2-arachidonoyglycerol (2-AG), both derived from arachidonic acid (Hansen and Diep, 2009; Serrano et al., 2012; Ueda et al., 2013). AEA and the rest of AEs are biosynthesized from glycerophospholipids through two steps. First, the generation of the precursor *N*-acyl-phosphatidylethanolamine (NAPE) by *N*-acyl transferase; and then, the release of the AE from NAPE by the NAPE-hydrolyzing phospholipase D (NAPE-PLD). After completing its physiological activity, AEs are reuptaken and degraded primarily through the fatty acid amide hydrolase (FAAH; Okamoto et al., 2009; Ueda et al., 2013; Pasquarelli et al., 2015). Regarding 2-AG, this MAG shares structural similarities with AEs but it is synthesized through a pathway involving different enzymes of synthesis and degradation. Thus, the main synthetic enzyme is the diacylglycerol lipase (DAGL), which produces 2-AG from arachidonic acid-containing diacylglycerol. 2-AG is later degraded primarily through the MAG lipase (MAGL) into glycerol and arachidonic acid (Ueda et al., 2013; Pasquarelli et al., 2015).

Other AEs such as palmitoylethanolamide (PEA) and oleoylethanolamide (OEA) share with AEA the same biosynthetic and degradative pathways, but they do not possess cannabinoid activity because they do not bind to cannabinoid receptors (Fu et al., 2003). However, PEA and OEA showed neuromodulatory properties as endogenous agonists of peroxisome proliferator-activated receptor alpha (PPARα). PPAR-α is a ligand-activated transcription factor, and it is part of the nuclear receptor superfamily. This receptor is expressed in microvascular, neuronal, and glial tissues and plays an important neuroprotective function in several diseases (Bordet et al., 2006; Bhateja et al., 2012; Moran et al., 2014).

Although the ECS has demonstrated to exert neuroprotective effects in models of neonatal and adult cerebral ischemia (Degn et al., 2007; Alonso-Alconada et al., 2011; Fernández-López et al., 2013; Esposito et al., 2014; England et al., 2015), these effects have never been tested in the model of PA developed by Bjelke et al. (1991). Moreover, there is no available information about the impact of PA on the expression and activity of the ECS/AEs system in the brain. In fact, OEA and PEA have shown neuroprotective effects in adult cerebral ischemia (Ahmad et al., 2012; Zhou et al., 2012; Yang et al., 2015), but they have never been tested in any of the neonatal hypoxia-ischemia models developed so far. Therefore, the main objective of this work was to analyze how the ECS/AEs signaling system is affected in the Bjelke’s rodent model of PA (Bjelke et al., 1991). To this purpose, we studied the expression of DAGLα, NAPE-PLD, CB1, PPARα, and FAAH in control (CTL), cesarean delivery (C+) and asphyctic (PA) 30-days-old rats. Because the dorsal hippocampus is one of the most vulnerable brain regions to hypoxic-ischemic injury, we focused our study in this area. In addition, we also studied the potential deleterious effect of PA on neuron and glial cells, and cognition.

## Materials and Methods

### Ethics Statement

Experimental procedures that required the use of animals were approved by: (1) the Institutional Animal Care and Use Committee of the School of Medicine at the University of Buenos Aires, (2) the Committee on Ethics of the Hospital R. U. of Malaga. In addition, the study was carried out following the European Directive 2010/63/EU on the protection of animals used for scientific purposes and Spanish regulations (RD 53/2013 and 178/2004). Every effort was made in order to reduce the number of animal employed and to minimize animal discomfort.

### Animals

Fifteen pregnant Sprague-Dawley rats were purchased from the School of Veterinary Sciences at the University of Buenos Aires. Seven days prior to delivery, pregnant rats were transported to our local *vivarium* and housed in individual cages in a controlled temperature (21 ± 2°C) and humidity (65 ± 5%) environment. A light/dark cycle of 12:12 h was employed with the light period beginning at 7 a.m. Food (Purina chow) and tap water were provided *ad libitum*. Seven pregnant rats were used as surrogate mothers while a group of eight pregnant rats was assigned to PA procedures. After weaning, the offspring was housed in groups of 3–4 animals of the same experimental condition. Different cohorts of animals were employed for behavioral assessment and immunohistochemical procedures.

### Cesarean Section and Perinatal Asphyxia Procedures

We employed the PA model developed by Bjelke et al. (1991), with minor modifications, as we previously described in Galeano et al. (2015). Ready-to-deliver pregnant rats (*n* = 8) “were left to deliver no more than two pups and were immediately euthanized by decapitation. Next, the uterus horns were rapidly isolated through an abdominal incision and one horn was opened, pups were removed, the amniotic fluid was cleaned, and the umbilical cord was ligated (cesarean section or C-section procedure). Concurrently, the remaining horn was placed in a water bath at 37°C for 19 min (moderate to severe PA). Afterward, the same procedures performed for the C-section were followed, but before ligation of the umbilical cord took place, pups were stimulated to breathe by performing tactile intermittent stimulation with pieces of medical wipes for a few minutes until regular breathing was established. This was unnecessary for pups born by C-section since they started breathing spontaneously.” Male pups that were born by C-section procedures (cesarean section group, C+) or by C-section procedures followed by asphyxia (PA) were left to recover under a heating lamp for ≈1 h. Female pups born by C-section or by C-section plus asphyxia were immediately euthanized by decapitation and only male pups were retained for all subsequent procedures and studies. “When the physiological conditions of the asphyxiated pups improved, C+ and PA pups were marked for identification and given to surrogate mothers which had delivered normally within the last 24 h.” Pups born by vaginal delivery from surrogate mothers were used as controls (control group, CTL). “A ≈95% of survival rate was observed in those pups that were born by C-section.” The survival rate dropped to ≈65% in the PA group. “In every case, we maintained litters of 10 pups with each mother.” All groups were equivalently represented in each litter.

### Behavioral Assessment

Ten rats from each group were submitted to a behavioral test battery at 30 days of age. Three days before the first behavioral test took place, rats were handled 5 min per day and weighed the last day. Behavioral tests were performed between 8:00 a.m. and 5:00 p.m. White noise was provided throughout testing. Training sessions were filmed using a digital camcorder (JVC Everio GZ-HD620) and later analyzed with a computerized video-tracking system (Ethovision XT, version 9, Noldus, Wageningen, The Netherlands) or with the ethological software JWatcher V1.0.

#### Novel Object Recognition Test

The Novel Object Recognition Test (NORT) is a behavioral test that assesses recognition memory (Ennaceur and Delacour, 1988; Ennaceur, 2010). The apparatus used was an open squared arena (60 cm × 60 cm × 40 cm) made of black melamine. Illumination was provided uniformly with a light intensity measured in the center of the arena of ≈70 lux. To habituate animals to the new environment, each rat was allowed to freely explore the apparatus during 10 min 1 day before the NORT was performed. The next day, NORT was carried out as described in Galeano et al. (2015): “rats were presented with two identical objects and allowed to explore them for 5 min (sample trial). Animals were returned to their cages during the inter-trial interval.” Four hours later, “one of the two familiar objects was replaced with a novel object and the rats were again allowed to explore them for 3 min (choice trial). Sets composed of three copies of the same object were used to prevent odor cues and all combinations and location of objects were used to prevent bias due to preference for a particular object or location. Exploration time was computed when the snout pointed to the object at a distance ≤2 cm. Discrimination index (d1) and discrimination ratio (d2) scores were calculated using the following formulas: d1 = tn – tf, and d2 = (tn – tf)/(tn + tf), where tn = the amount of time rats explored the novel object and tf = the amount of time rats explored the familiar object.” It is important to note that in the present study the inter-trial interval was longer (4 h) than the one used (1 h) in the previous study by Galeano et al. (2015).

#### Morris Water Maze Test

The apparatus was the same used in previous studies (Galeano et al., 2014, 2015) consisting “of a circular black galvanized steel tank (180 cm in diameter and 60 cm deep). The tank was filled to a depth of 36–40 cm with water at 22 ± 1°C. The maze was divided into four imaginary quadrants (A, B, C, and D) and a circular platform, made of transparent acrylic, was placed 2 cm above (visible escape platform) or beneath the water surface (hidden escape platform), in the center of one of the quadrants (35 cm from the edge of the tank). To enhance the visibility of the platform during the cued learning training, a “flag” was attached to the platform. Four starting positions were established according to the four quadrants (a, b, c, and d). To provide external reference points, multiple extra-maze visual cues of different shapes and sizes were hung on the wall of the experimental room. Indirect illumination was provided by four spiral compact fluorescent lamps in each corner facing the walls.” Variables registered were: latency to find the escape platform and swimming speed. During the probe trial, the percentage of time spent in each quadrant and the number of crossings over the previous platform location were also registered.

##### Cued learning

The cued learning was conducted exactly as previously described by Galeano et al. (2015): “During cued learning the platform protruded 2 cm above water surface and a “flag” was attached to it (visible escape platform). The maze was surrounded by black curtains to minimize the availability of extra-maze cues. For each of the four trials conducted on each day, the platform was moved to a different quadrant and a different start location was used. If a rat had not located the platform before 120 s elapsed, it was gently guided to the platform location and was allowed to remain there for 15 s. Inter-trial interval duration was approximately 30 s. Two days of cued training were conducted.”

##### Spatial learning and reference memory

Procedures employed were previously described by Galeano et al. (2015): “Briefly, the spatial learning task was conducted over five consecutive days with four trials per day. During each trial, a rat was gently released into the tank from one of the four starting positions and it was able to escape from the water using the hidden escape platform that was kept in the same location throughout the five sessions of the spatial learning task. A trial was finished when the rat found the escape platform or when 120 s had elapsed, whichever occurred first. If a rat failed to find the platform, the experimenter guided the animal to it. Rats remained on the platform for 15 s. Inter-trial interval duration was approximately 30 s. In each session, the four starting positions were used and the order of the sequence was changed pseudo-randomly between days. Twenty-four hours after the last trial of the spatial learning task, reference memory was assessed with a probe trial of 60 s in which the escape platform was removed from the tank and each rat was released from a new starting position not used during the spatial learning task. Time spent in each quadrant was recorded. When sessions finished rats were dried and returned to their home cage.” In addition, platform crossings were also recorded as an additional measure to assess animal performance during the probe trial.

### Tissue Processing

At 30 days of age, rats were anesthetized with 28% (w/v) chloral hydrate (0.1 ml/100 g of body weight). Next, animals were intracardially perfused with 4% formaldehyde (freshly made from paraformaldehyde; Sigma-Aldrich, St. Louis, MO, USA) in 0.1 M phosphate buffer (pH 7.4). Subsequently, rats’ brains were dissected and post-fixed during 2 h in the formaldehyde solution. Finally, rats’ brain were stored in 0.1 M PBS (pH 7.3) containing 30% sucrose and 0.01% sodium azide (NaN_3_) at 4°C until further processing. Finally, 30-μm-thick coronal sections were obtained using a freezing microtome and stored in PBS with 0.002% (w/v) NaN_3_ at 4°C until immunohistochemistry procedures were carried out.

### Immunohistochemistry

We assessed the immunohistochemical expression of NeuN, glial fibrillary acidic protein (GFAP), DAGLα, NAPE-PLD, CB1, PPARα, and FAAH in the dorsal hippocampus. For this purpose, 30 μm free floating sections were first washed with 0.1 M PBS (pH 7.3), and then incubated in 50 mM citrate buffer (pH 6) for 30 min at 80°C. Next, sections were washed three times in PBS (0.1 M, pH 7.3). In order to inactivate the endogenous peroxidase, sections were then incubated in 3% H_2_O_2_ and 10% methanol in PBS (0.1 M) for 20 min at room temperature. Following, sections were washed again three times in PBS (0.1 M, pH 7.3) and blocked with 10% donkey or goat serum diluted in PBS (containing 0.2% Triton X-100). Next, free floating sections were incubated overnight with the corresponding primary antibody at 4°C [mouse anti-NeuN (1:500, MAB377, Millipore); mouse anti-GFAP (1:500, G3893, Sigma), rabbit anti-DAGLα (1:250, developed by our group); guinea pig anti-NAPE-PLD (1:500, Frontier Institute); rabbit anti-PPARα (1:100, P11120812, Fitzgerald); rabbit anti-CB1 (1:200, Frontier Institute) and rabbit anti-FAAH (1:200, 157878, Cayman); Rivera et al., 2014a,b; procedures carried out to develop polyclonal antibody against DAGLα were described in detail by Suárez et al., 2008]. The next day, sections were again washed three times in PBS (0.1 M, pH 7.3) and then incubated for 1 h at room temperature with the corresponding biotinylated secondary antibody in a 1:500 dilution(goat anti-mouse IgG, 125K6063, Sigma; donkey anti-rabbit IgG, 5356499, GE Healthcare; goat anti-guinea pig, W0726, Vector Laboratories; Rivera et al., 2014a,b). Next, sections were washed other three times in PBS (0.1 M, pH 7.3) and incubated with the ExtrAvidin peroxidase complex (1:2000, Sigma, USA) for 1 h in the dark. Following three washes in PBS, immunolabeling was revealed exposing sections to 0.05% diaminobenzidine (Sigma, USA) and 0.03% H_2_O_2_ in PBS (in the case of CB1 and PPARα, nickel-DAB enhancement procedure was carried out with the addition of 0.05% nickel ammonium sulfate). The reaction was stopped using PBS and tissues were then washed several times in PBS. To avoid variations in the intensity of staining due to procedure, including potential differences in the time that sections are exposed to DAB, batches (each one containing approximately the same number of sections from the three experimental groups) stained with the same primary antibody were run simultaneously. In each batch, negative controls were obtained by omitting primary antibody and no background staining was observed for any of the secondary antibodies employed (see Supplementary Material). Next, glass slides, which were treated with poly-L-lysine solution (Sigma, USA), were employed to mount the free floating sections. Afterward, mounted sections were dehydrated in ascending ethanol concentrations (50-70-96-100%), cleared with xylene and coverslipped using Eukitt mounting medium (Kindler GmBH& Co, Freiburg, Germany). Digital photomicrographs were taken with a 10x objective (or 40x for insets) using the same conditions of light and brightness/contrast employing an Olympus BX41 microscope coupled with an Olympus DP70 digital camera (Olympus, Germany) or an Olympus BX60 microscope coupled to Olympus DP71 digital camera (Olympus, USA). Total magnification was 200x for those photomicrographs taken with a 10x objective and 800x for those taken with a 40x objective (insets).

### Immunostaining Quantification

We examined 5–7 coronal sections obtained from Bregma levels: -3.14 to -4.30 mm (dorsal hippocampus; Paxinos, 2007), in a total of 5–6 animals per group. We focused on *cornu ammonis* 1 (CA1), *cornu ammonis* 3 (CA3), and dentate gyrus (DG). In every section, immunoreactivity, or the number of positive cells, was quantified in both hemispheres and averaged. From each region and animal a mean was calculated and used for subsequent statistical analysis. Percentage of reactive area (NeuN, NAPE-PLD, and FAAH), densitometry (DAGLα, NAPE-PLD, and CB1) and cell counting (GFAP and PPARα) were performed using Image J 1.38X (NIH, USA). We used percentage of reactive area in order to determine the tissue area that was immunoreactive. Images were changed to binary code and the positive staining was used to set the threshold, the optical density was automatically determined and the percentage of stained area was calculated over the total area of the frame which was for all cases of 20060 μm^2^ (170 μm × 118 μm). In order to measure levels of those markers that present diffuse staining (DAGLα, CB1), we used densitometric quantification (Lopez-Rodriguez et al., 2014). Briefly, each image was changed to binary code, the area of interest was delineated by the operator, and the optical density was automatically calculated. For GFAP and PPARα, we determined the number of cells per area using the “Cell Counter” Image J plugin tool. In photomicrographs of sections stained with GFAP containing CA1, CA3 or DG, we set 0.08 mm^2^ squares distributed so that the whole section areas were represented. For PPARα, we set 0.02 mm^2^ squares along the *stratum pyramidale* (sp) of CA1 and CA3 and the granular cell layer (gcl) of DG, in such a manner that the whole layers were totally represented. Subsequently, we manually determined the number of cells in each square and calculated the number of cells per mm^2^. Each quantification was made without knowledge of the experimental groups coding the samples by an independent experimenter.

### Statistical Analysis

Results were shown as the mean ± SEM. Data were analyzed by either one-way, two-way, or two-way mixed ANOVA tests followed by Tukey’s *post hoc* tests for multiple comparisons (in the case of behavioral data) or by Bonferroni’s correction for multiple comparisons (for immunohistochemical analysis), unless noted otherwise. When normality and/or homoscedasticity were not met, non-parametric tests were carried out (Kruskal–Wallis followed by Mann–Whitney tests). A probability equal or less than 5% was considered significant (two-tailed). SSPS 15.0 for windows (SPSS, Inc., USA) were used to perform statistical analyses.

## Results

### Novel Object Recognition Test

**FIGURE 1:**
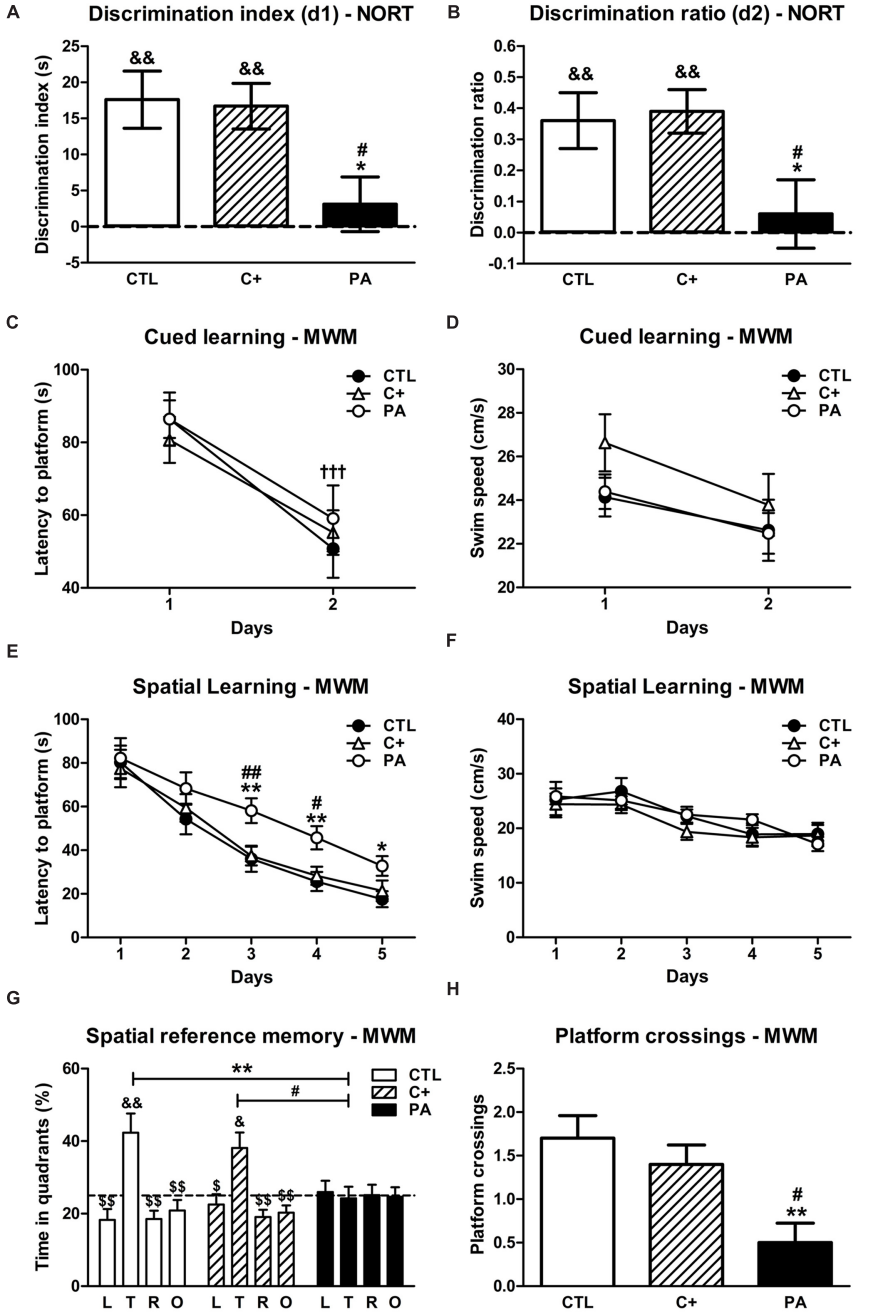
**Behavioral assessment. (A)** Discrimination indexes and **(B)** discrimination ratios of the CTL, C+ and PA groups in the choice trial of the NORT. ^∗^*p* < 0.05 vs. CTL; ^#^*p* < 0.01 vs. C+; ^&&^*p* < 0.01 vs. scores expected by chance (dashed lines). **(C)** Latencies to platform and **(D)** swimming speeds of the different experimental groups during the cued learning phase of the MWM. ^†††^*p* < 0.001 vs. day 1 for all the groups. **(E)** Latencies to platform and **(F)** swimming speeds of the different experimental groups during the spatial learning phase of the MWM. ^∗^*p* < 0.05 vs. CTL; ^∗∗^*p* < 0.01 vs. CTL; ^#^*p* < 0.05 vs. C+; ^##^*p* < 0.01 vs. C+. **(G)** Percentages of time spent in the four quadrants and **(H)** number of platform crossings during the probe trial. ^∗∗^*p* < 0.01 vs. the percentage of time spent in the target quadrant by the PA group **(G)** or vs. CTL **(H)**; ^#^*p* < 0.05 vs. the percentage of time spent in the target quadrant by the PA group **(G)** or vs. C+ **(H)**; ^$^*p* < 0.05 and ^$$^*p* < 0.01 vs. the percentage of time spent in the target quadrant within the same group; ^&^*p* < 0.05 and ^&&^*p* < 0.01 vs. the percentage of time expected by chance (25%; dashed line). CTL, control group; C+, cesarean section group; PA, perinatal asphyxia group; NORT, Novel Object Recognition Test; MWM, Morris water maze test; L, left quadrant; T, target quadrant; R, right quadrant; O, opposite quadrant.

One-way ANOVA tests carried out on d1 and d2 showed that the main effect of birth condition (CTL, C+, and PA) was significant in both cases [*F*_(2,29)_ = 4.93, *p* < 0.05; *F*_(2,29)_ = 4.15, *p* < 0.05, respectively]. *Post hoc* multiple comparisons revealed that d1 and d2 scores were significantly lower in the PA group than in CTL and C+ groups (*p* < 0.05 for all the comparisons, see **Figures 1A,B**). Moreover, one sample *t*-tests showed that d1 and d2 scores were significantly different from those expected by chance in CTL and C+ groups (*p* < 0.01 for all cases; see **Figures 1A,B**), but not in the PA group (*p* = n.s. for both scores; see **Figures 1A,B**). These results indicated that PA rats explored both objects, i.e., the novel and the familiar, a similar amount of time during the choice trial, and thus it could be concluded that recognition memory was impaired in this group of rats.

### Morris Water Maze Test

#### Cued Learning

Two-way mixed ANOVA test indicated that the main effect of day was significant [*F*_(1,27)_ = 75.42, *p* < 0.001]. The main effect of birth condition and the interaction birth condition × day were not (*F* < 1 in both cases). These results indicated that all groups diminished their escape latency to reach the visible platform from day 1 to 2 (see **Figure 1C**). This drop in escape latency was not due to a decrease in swimming speed since none of the groups showed a reduction in the latter from first to second day (*F* < 1 for the main effects of day and birth condition and for the interaction day × birth condition; see **Figure 1D**). A similar performance of groups in the cued learning suggested that they did not have a deficit in their swimming abilities and were visually unimpaired.

#### Spatial Learning

To assess the performance of groups in the spatial learning phase of the Morris water maze test, we carried out two-way mixed ANOVA tests with birth condition as between-subject factor and day as the within-subject factor. When the dependent variable latency to platform was analyzed, results showed that the main effect of day was significant [*F*_(4,108)_ = 123.17, *p* < 0.001], while the main effect of birth condition and the interaction birth condition × day were not [*F*_(2,27)_ = 2.25, *p* = n.s.; *F*_(8,108)_ = 1.54, *p* = n.s., respectively]. Although the interaction did not reach significance, Tukey’s *post hoc* tests for multiple comparisons indicated that, on days 3 and 4, the PA group showed significantly higher latencies to reach the hidden platform than CTL and C+ groups (*p* < 0.05 or *p* < 0.01 for each comparison; see **Figure 1E**). During the last day of the spatial learning phase, the PA group displayed a higher latency to reach the hidden platform than the CTL group (*p* < 0.05, see **Figure 1E**), but the latency was not statistically different from that showed by the C+ group (*p* = n.s., see **Figure 1E**). These results indicated that the PA group showed a deficit in the spatial learning phase of the water maze. Regarding swimming speed, the two-way mixed ANOVA test showed that the main effect of day was significant [*F*_(4,108)_ = 12.71, *p* < 0.001], while the main effect of birth condition and the interaction birth condition × day were not (*F* < 1 for both cases). Furthermore, any of the *post hoc* tests reached statistical significance (see **Figure 1F**). These results strongly suggested that the learning deficit showed by the PA group could not be better explained by differences in swimming abilities and/or motivation to complete the task.

#### Spatial Reference Memory

To analyze the percentage of time spent in each quadrant during the probe trial, a two-way mixed ANOVA test, with birth condition as between-subject factor and quadrant as within-subject factor, was computed. Results showed that the main effect of quadrant and the interaction quadrant × birth condition were significant [*F*_(3,81)_ = 9.89, *p* < 0.001; *F*_(6,81)_ = 3.10, *p* = 0.01, respectively], while the main effect of birth condition was not significant (*F* < 1). Tukey’s *post hoc* tests for multiple comparisons revealed that the CTL and C+ groups spent significantly greater percentages of time in the quadrant where the platform was located during the spatial learning trials (target quadrant) than in any of the other quadrants (*p* < 0.05 or *p* < 0.01 for each comparison, see **Figure 1G**). On the contrary, the PA group spent similar percentages of time in all quadrants (*p* = n.s. for all the comparisons, see **Figure 1G**). In addition, CTL and C+ groups spent significantly greater percentages of time in the target quadrant than the PA group (*p* < 0.01 and *p* < 0.05, respectively; see **Figure 1G**). When we carried out one sample *t*-tests to compare the percentages of time that each group spent in each quadrant with the percentage of time expected by chance (25%), results showed that CTL and C+ groups spent significantly greater time in the target quadrant than that expected by chance (*t* = 3.27, *df* = 9, *p* = 0.01; *t* = 3.08, *df* = 9, *p* < 0.05, respectively), while the PA group did not (*t* = -0.24, *df* = 9, *p* = n.s.). Finally, we analyzed the number of times that rats crossed the area where the platform had been located during the spatial learning trials, as an additional indicator of the performances of animals in the probe trial. Kruskal–Wallis test revealed that groups differed in the number of platform crossings (*H* = 9.84, *p* < 0.01). *Post hoc* Mann–Whitney tests, with Bonferroni’s adjustment, showed that the PA group crossed the former platform location significantly less times than CTL and C+ groups (*U* = 14, *p* < 0.01; *U* = 19.5, *p* < 0.05, respectively; see **Figure 1H**). Overall, these results indicated that the PA group did not exhibit a searching bias toward the target quadrant and the former platform location, and thus, this group presented a spatial reference memory impairment.

### NeuN and GFAP Immunostaining in Dorsal Hippocampus

**FIGURE 2:**
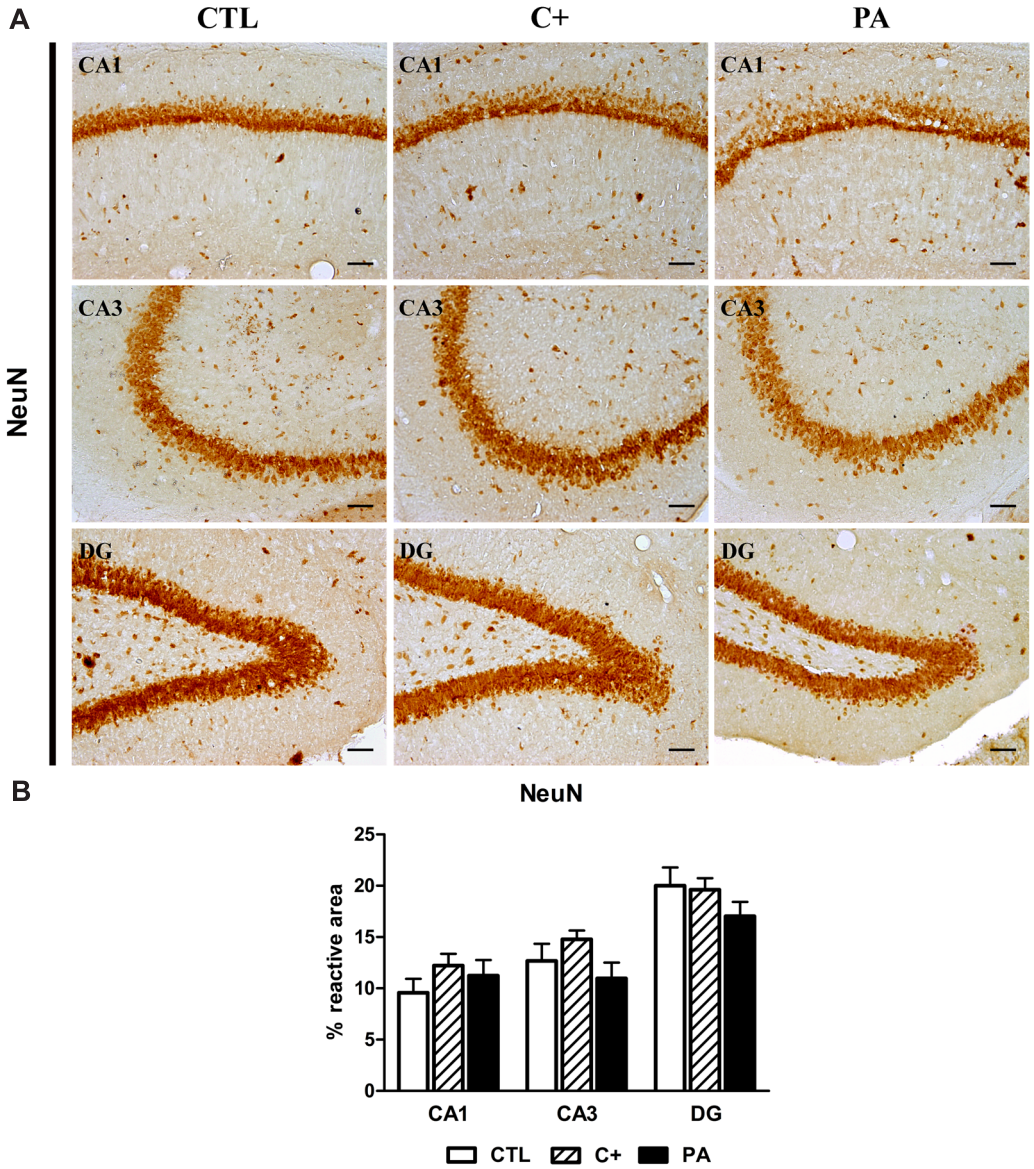
**NeuN immunostaining in dorsal hippocampus. (A)** Photomicrographs of NeuN-positive cells in CA1, CA3, and DG regions of the dorsal hippocampus in CTL, C+ and PA groups. Scale bars = 15 μm. **(B)** Percentage of NeuN immunopositive area in CA1, CA3, and DG regions of the dorsal hippocampus in all the experimental groups. Bars and error bars show the mean + SEM of 5–7 observations per animal of a total of six rats per group. CTL, control group; C+, cesarean section group; PA, perinatal asphyxia group; CA1, *cornu ammonis* 1; CA3, *cornu ammonis* 3; DG, dentate gyrus.

**FIGURE 3:**
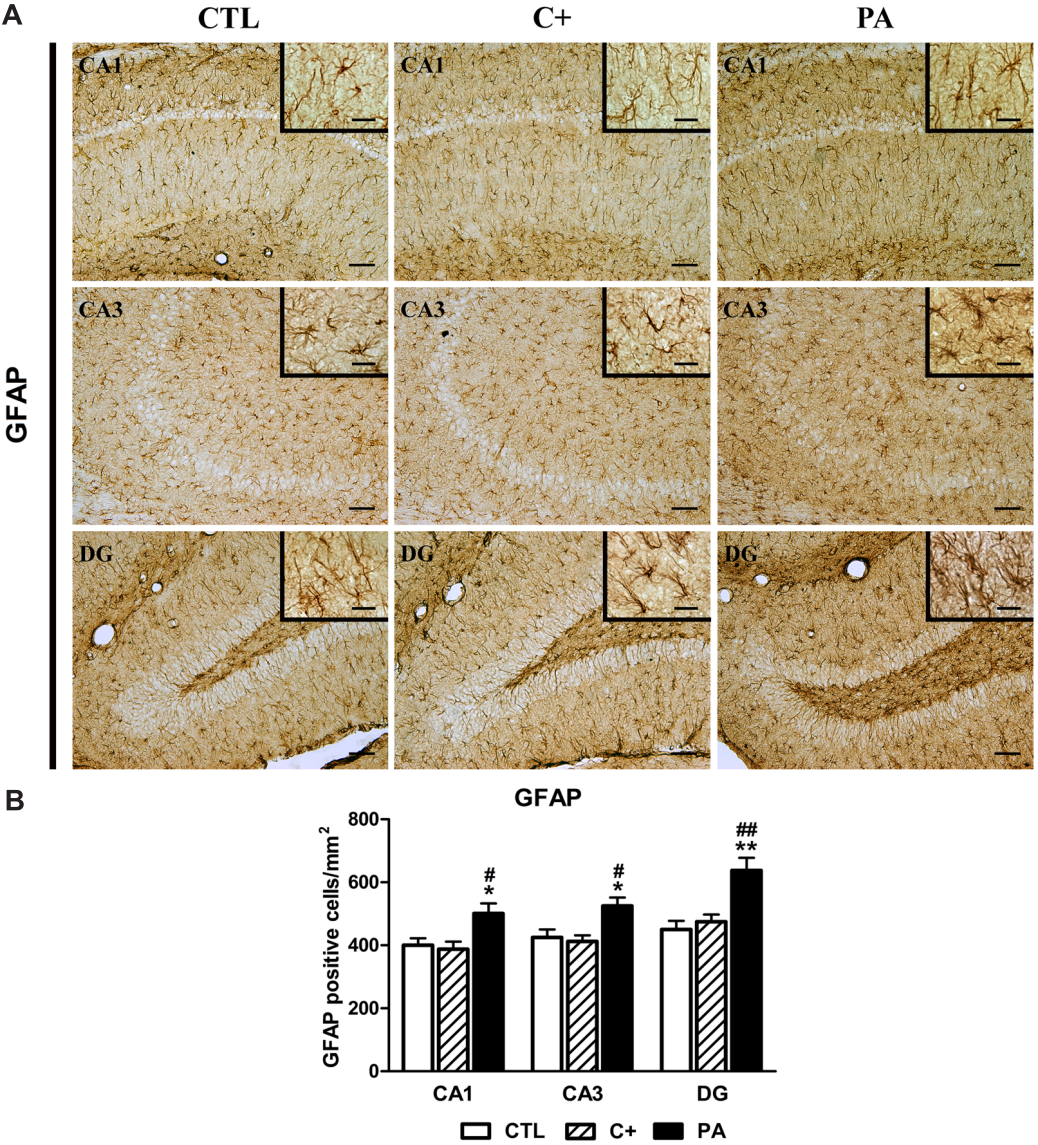
**Glial fibrillary acidic protein (GFAP) immunostaining in dorsal hippocampus. (A)** Representative photomicrographs showing GFAP-positive cells in CA1, CA3, and DG regions of the dorsal hippocampus in the different experimental groups. Scale bars = 15 and 3 μm in insets. **(B)** Quantification of the number of GFAP-positive cells in CA1, CA3, and DG regions of the dorsal hippocampus in the different experimental groups. Bars and error bars show the mean + SEM of 5–7 observations per animal of a total of six rats per group. ^∗^*p* < 0.05 vs. CTL; ^∗∗^*p* < 0.01 vs. CTL; ^#^*p* < 0.05 vs. C+; ^##^*p* < 0.01 vs. C+. CTL, control group; C+, cesarean section group; PA, perinatal asphyxia group; CA1, *cornu ammonis* 1; CA3, *cornu ammonis* 3; DG, dentate gyrus.

One-way ANOVA tests showed that the percentage of reactive area for NeuN did not differ between groups, either in CA1, CA3, or DG regions of the dorsal hippocampus [CA1: *F* < 1; CA3: *F*_(2,15)_ = 1.87, *p* = n.s.; DG: *F*_(2,15)_ = 1.24, *p* = n.s.; see **Figures 2A,B**]. When analyzing the number of GFAP-positive cells, we found that groups differed between them in the studied regions of the dorsal hippocampus [CA1: *F*_(2,15)_ = 5.52, *p* < 0.05; CA3: *F*_(2,15)_ = 6.57, *p* < 0.01; DG: *F*_(2,15)_ = 10.71, *p* < 0.01]. *Post hoc* multiple comparison tests revealed that the PA group showed an increased number of GFAP-positive cells compared to those measured in CTL and C+ groups in the three hippocampal regions (*p* < 0.05 or *p* < 0.01 for all the comparisons; see **Figures 3A,B**).

### Immunohistochemical Expression of DAGLα and NAPE-PLD in Dorsal Hippocampus

**FIGURE 4:**
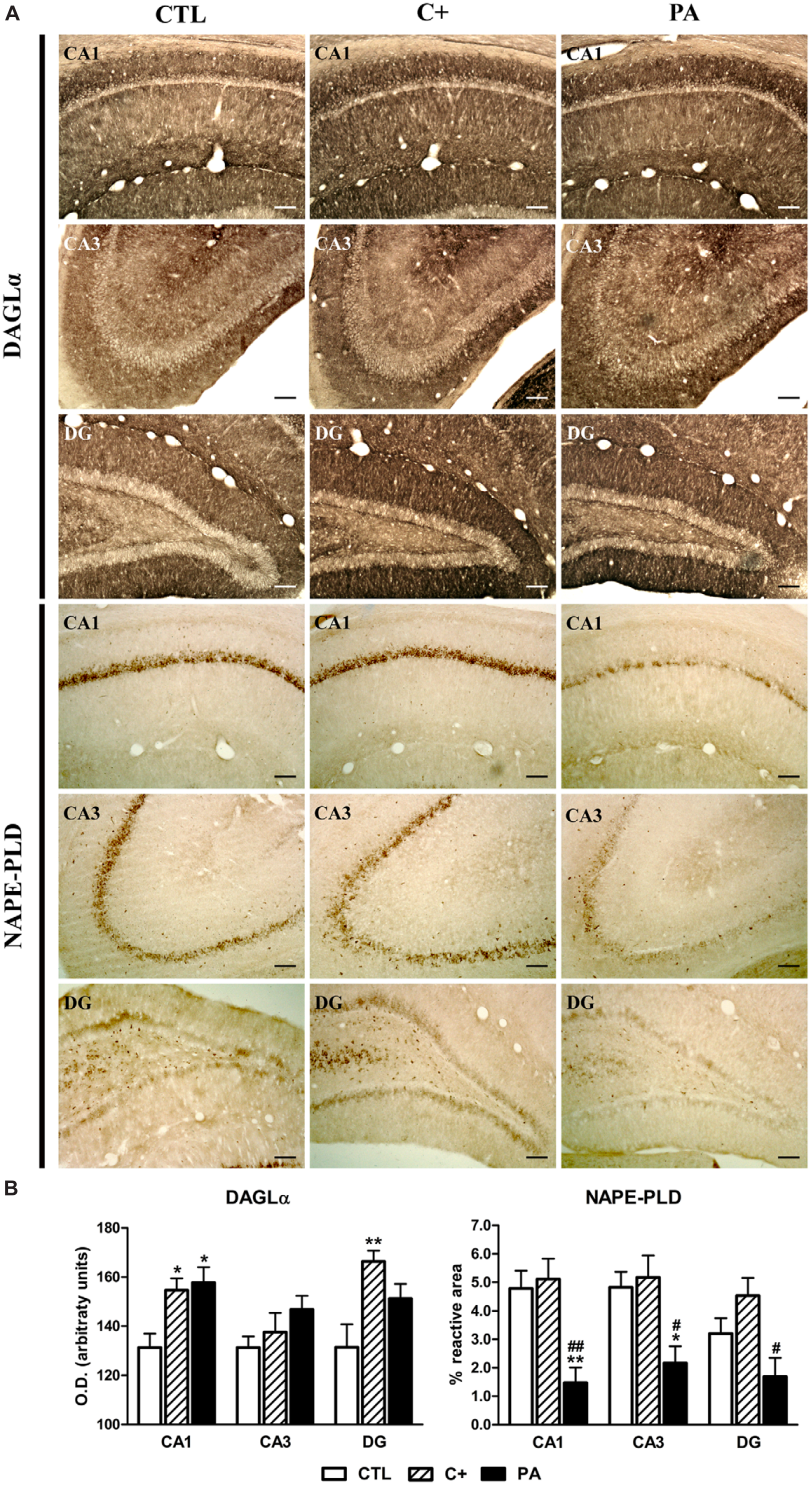
**Immunohistochemical expression of DAGLα and NAPE-PLD in dorsal hippocampus. (A)** Photomicrographs showing DAGLα (upper panel) and NAPE-PLD (lower panel) immunostaining in CA1, CA3, and DG regions of the dorsal hippocampus in the different experimental groups. Scale bars = 17 μm. **(B)** Immunostaining quantification of DAGLα (left panel) and NAPE-PLD (right panel) in CA1, CA3, and DG regions of the dorsal hippocampus in all groups. DAGLα immureactivity was measured by densitometry while NAPE-PLD positive immunostaining was quantified as the percentage of the stained area over the total area of the region of interest. Bars and error bars show the mean + SEM of 5–7 observations per animal of a total of six rats per group. ^∗^*p* < 0.05 vs. CTL; ^∗∗^*p* < 0.01 vs. CTL; ^#^*p* < 0.05 vs. C+; ^##^*p* < 0.01 vs. C+. CTL, control group; C+, cesarean section group; PA, perinatal asphyxia group; CA1, *cornu ammonis* 1; CA3, *cornu ammonis* 3; DG, dentate gyrus.

One-way ANOVA tests showed that the mean optical densities of DAGLα immunostaining were different between groups in CA1 and DG regions [*F*_(2,15)_ = 6.57, *p* < 0.01; *F*_(2,15)_ = 6.60, *p* < 0.01, respectively], while no statistically significant differences were observed in the CA3 region [*F*_(2,15)_ = 1.61, *p* = n.s.]. *Post hoc* pairwise comparison tests indicated that C+ and PA groups showed significantly higher mean optical densities than the CTL group in the CA1 region (*p* < 0.05 for both cases; see **Figure 4A**, upper panel, and **4B**, left panel). In the DG region, the C+ group displayed a higher mean optical density than the CTL group (*p* < 0.01; see **Figure 4A**, upper panel, and **4B**, left panel), while the mean optical density detected in the PA group was not statistically different than those detected in the CTL and C+ groups (*p* = n.s. for both comparisons; see **Figure 4A**, upper panel, and **4B**, left panel). Regarding the immunohistochemical expression of NAPE-PLD, one-way ANOVA tests showed that the percentages of reactive area were statistically different between groups in the studied hippocampal regions [CA1: *F*_(2,15)_ = 10.16, *p* < 0.01; CA3: *F*_(2,15)_ = 6.58, *p* < 0.01; DG: *F*_(2,15)_ = 5.45, *p* < 0.05]. *Post hoc* multiple comparison tests revealed that PA group showed a lower percentage of reactive area in CA1 and CA3 regions than the ones measured in CTL and C+ groups (*p* < 0.05 or *p* < 0.01 for all the comparisons; see **Figure 4A** lower panel and **4B**, right panel). In the DG, the PA group showed a lower percentage of reactive area than that measured in the C+ group (*p* < 0.05; see **Figure 4A**, lower panel and **4B**, right panel), but no statistically significant differences were found between PA and CTL groups (*p* = n.s.; see **Figure 4A**, lower panel and **4B**, right panel).

### Immunohistochemical Expression of CB1 and PPARα in Dorsal Hippocampus

**FIGURE 5:**
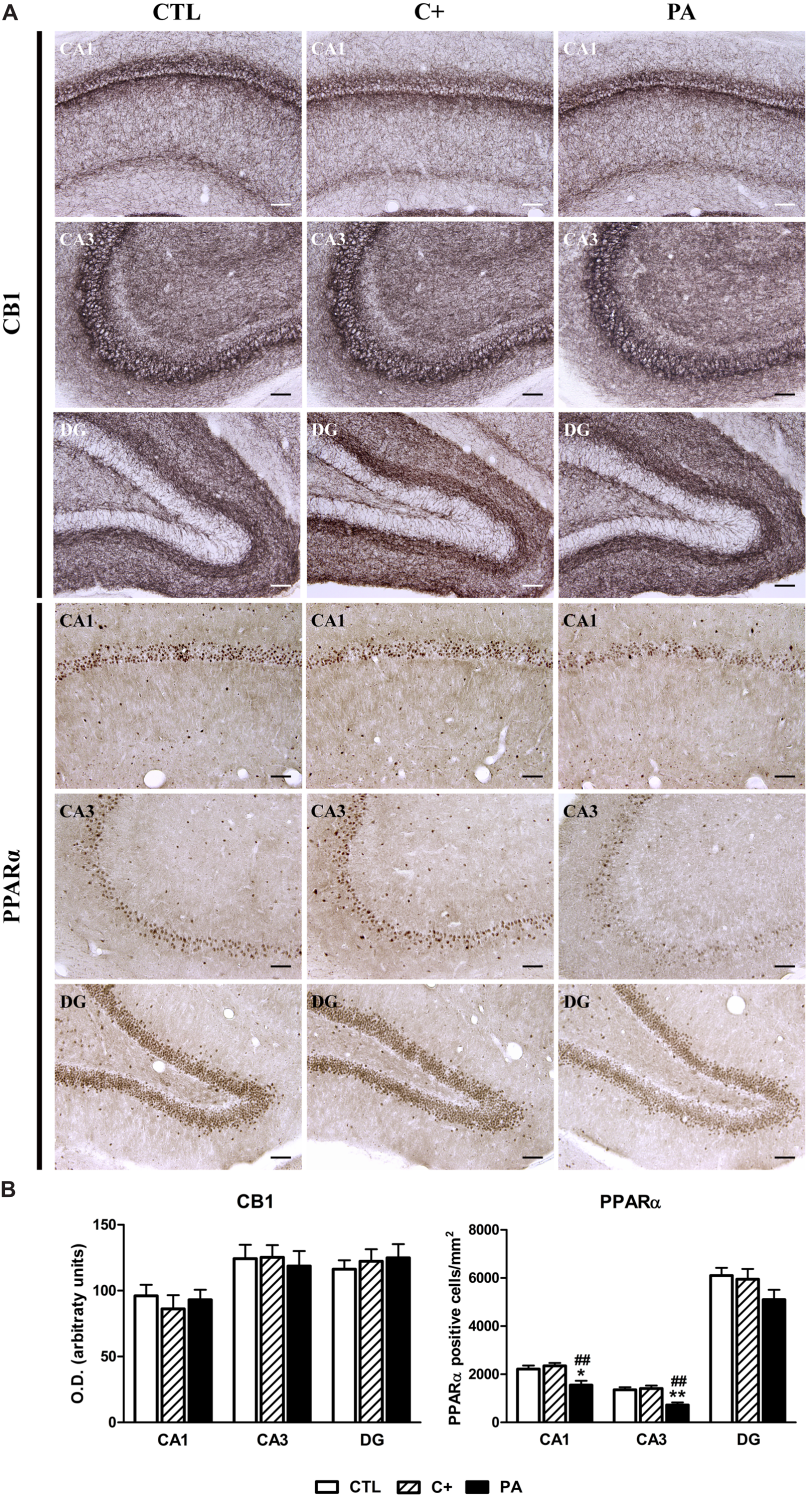
**Immunohistochemical expression of CB1 and PPARα in dorsal hippocampus. (A)** Photomicrographs of CB1 (upper panel) and PPARα (lower panel) immunostaining in CA1, CA3, and DG regions of the dorsal hippocampus in CTL, C+, and PA groups. Scale bars = 15 μm. **(B)** Densitometric quantification of CB1 (left panel) and number of PPARα positive nuclei per mm^2^ (right panel) in the *Stratum pyramidale* of the CA1 and CA3 areas, and in the granular cell layer of the DG region, in the different experimental groups. Bars and error bars show the mean + SEM of 5–7 observations per experimental subject of a total of 5–6 animals per group. ^∗^*p* < 0.05 vs. CTL; ^∗∗^*p* < 0.01 vs. CTL; ^##^*p* < 0.01 vs. C+. CTL, control group; C+, cesarean section group; PA, perinatal asphyxia group; CA1, *cornu ammonis* 1; CA3, *cornu ammonis* 3; DG, dentate gyrus.

The analysis of the immunohistochemical expression of CB1 in dorsal hippocampus revealed that there are no significant differences between groups in none of the quantified regions (*F* < 1 for CA1, CA3, and DG; see **Figure 5A**, upper panel, and **5B**, left panel). The number of PPAR-positive cells differed between groups in the CA1 and CA3 regions [*F*_(2,15)_ = 7.94, *p* < 0.01; *F*_(2,15)_ = 11.38, *p* < 0.01, respectively], while no differences were observed in the DG [*F*_(2,15)_ = 1.92, *p* = n.s.]. *Post hoc* pairwise comparison tests showed that, in the PA group, the number of PPAR-positive cells was significantly lower compared to those measured in CTL and C+ groups (*p* < 0.05 or *p* < 0.01 for all the comparisons; see **Figure 5A**, lower panel, and **5B**, right panel).

### Immunohistochemical Expression of FAAH in Dorsal Hippocampus

**FIGURE 6:**
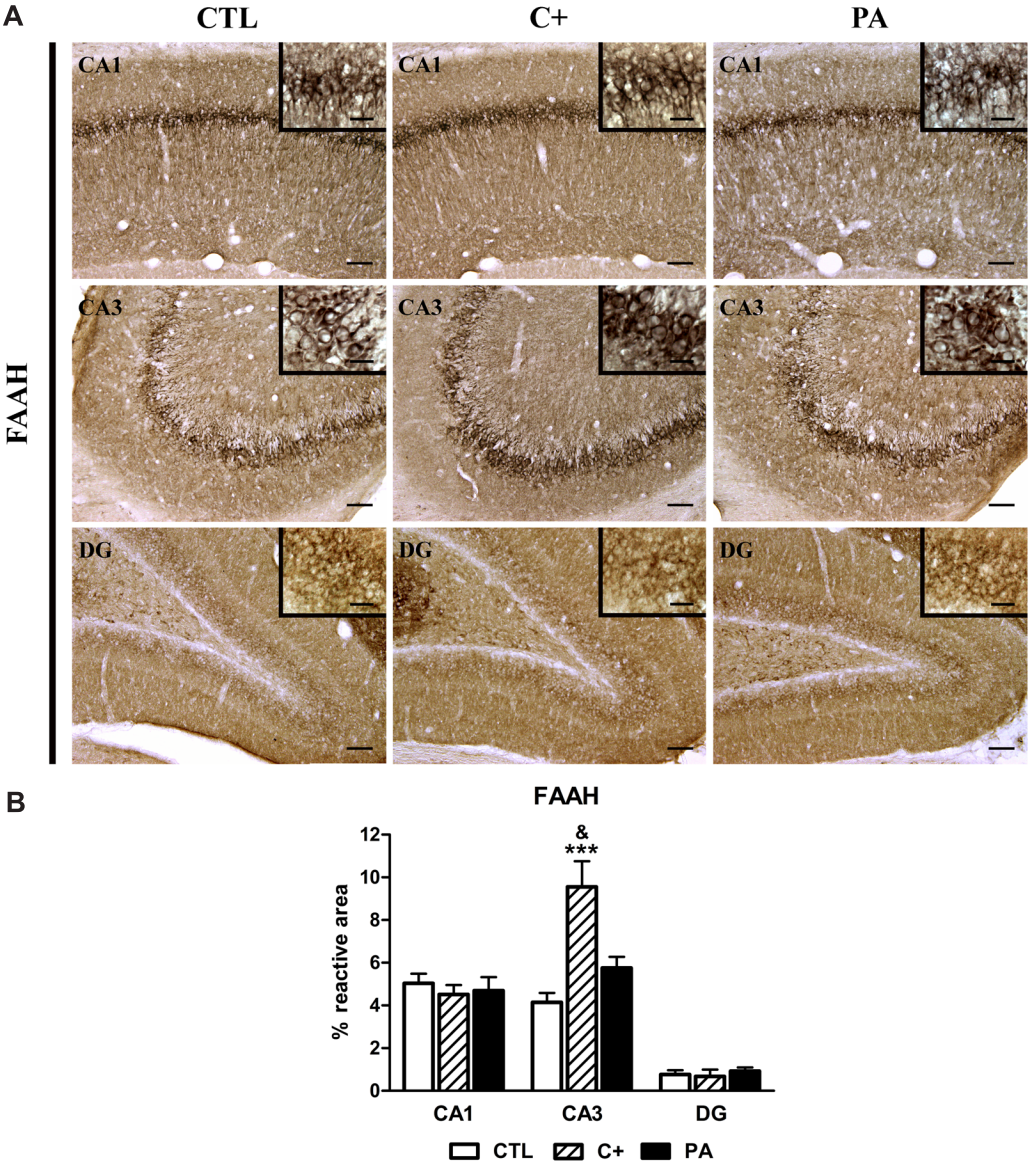
**Immunohistochemical expression of FAAH in dorsal hippocampus. (A)** Representative photomicrographs showing positive immunostaining of FAAH in CA1, CA3, and DG regions of the dorsal hippocampus in the different groups (CTL, C+, and PA). Scale bars = 15 and 3 μm in insets. **(B)** Quantification of FAAH, as the percentage of stained area over the total area of the region of interest, in CA1, CA3, and DG regions of the dorsal hippocampus in the different experimental groups. Bars and error bars show the mean + SEM of 5–7 observations per rat of a total of six animals per group. ^∗∗∗^*p* < 0.001 vs. CTL; ^&^*p* < 0.05 vs. PA. CTL, control group; C+, cesarean section group; PA, perinatal asphyxia group; CA1, *cornu ammonis* 1; CA3, *cornu ammonis* 3; DG, dentate gyrus.

The percentages of reactive area only differed between groups in the CA3 region [CA1: *F* < 1; CA3: *F*_(2,15)_ = 12.13, *p* < 0.001; DG: *F* < 1]. *Post hoc* multiple comparison tests indicated that the C+ group showed a significantly higher percentage of reactive area than that observed in the CTL and PA groups in the CA3 region (*p* < 0.001 and *p* < 0.05, respectively; see **Figures 6A,B**).

## Discussion

In last years, the ECS has received growing attention as new evidences show it plays a fundamental role in a myriad of brain processes, including synaptic plasticity, neurogenesis, learning and memory, mood regulation, reward processes, central control of feeding behavior, among others (Bermudez-Silva et al., 2010; Cachope, 2012; Mechoulam and Parker, 2013). Moreover, the modulation of the ECS seems to exert neuroprotective actions in several models of neonatal hypoxia-ischemia and adult cerebral ischemia (Degn et al., 2007; Alonso-Alconada et al., 2011; Fernández-López et al., 2013; Esposito et al., 2014; England et al., 2015). For instance, endocannabinoids (AEA and 2-AG) were demostrated to reduce brain injury in preweaning or adult rats that were submitted to a hypoxic-ischemic episode or an excitotoxic brain lesion during the neonatal period (Shouman et al., 2006; Carloni et al., 2012; Lara-Celador et al., 2012). The CB1/CB2 receptor agonist, WIN-55212, has also showed to exert neuroprotective actions in neonatal hypoxia-ischemia, preventing early and delayed neuronal death (Martínez-Orgado et al., 2003). The prevention of delayed neuronal death was mediated through CB1-dependent mechanisms, because the co-administration of the cannabinoid CB1 receptor antagonist SR141716 abolished the WIN-55212-induced prevention effect (Martínez-Orgado et al., 2003). In the case of AEs devoid of cannabinoid activity, Zhou et al. (2012) have demonstrated that the administration of OEA previous to the induction of ischemic brain injury reduced infarct volume and alleviated brain edema in adult mice. This neuroprotective effect was mediated by the increased expression of PPARα induced by the OEA administration (Zhou et al., 2012). Interestingly, in the present study we found a reduced expression of NAPE-PLD, an enzyme that mediates OEA synthesis, along with a reduction of the expression of OEA receptor PPARα, suggesting that the potential neuroprotective role of this system is impaired.

### Asphyctic Rats Show Cognitive Impairments Associated with Astrogliosis in the Hippocampus in the Absence of Neuronal Loss

At a behavioral level, the results of this study indicate that PA is associated with impairments in recognition memory, spatial learning, and reference memory in the Morris water maze (**Figures 1A–H**). In previous works, we demonstrated that 3- and 18-months-old rats that were submitted to PA also display spatial learning and reference memory deficits in the Morris water maze (Galeano et al., 2011, 2015). With the addition of these results, we can conclude that spatial and reference memory impairments are presented as early as 30 days after PA and prevail, at least, until middle age. In relation to recognition memory, we were not previously able to detect an impairment in this kind of memory in 18-months-old asphyctic rats (Galeano et al., 2015). As it was discussed in the cited study, 1 h of inter-trial interval between sample and choice trials could be too short to reveal group differences. In this study, we extended the inter-trial interval to 4 h and we were able to detect a recognition memory impairment in the PA group. Another important difference, between the present and the previous study, is the age of the animals. Young adult asphyctic rats (1-month-old) were employed in the present study while middle-aged asphyctic rats (18-months-old) were used in the previous one. Middle-aged control rats might have subtle deficits in recognition memory that could make more difficult to reveal statistically significant differences when compared with asphyctic group.

These cognitive deficits were somehow expected because both behavioral tests are hippocampal-dependent tasks (Clark et al., 2000; Vorhees and Williams, 2014), and the hippocampus is one of the most affected structures by PA, both in animal models and humans (Vannucci, 1990; Kasdorf et al., 2014). In fact, we observed a strong astroglial reaction in the hippocampus of asphyctic animals (see **Figures 2A,B**, left panel). This astroglial reaction was not accompanied by neuronal loss as it was previously reported by our group in this model of PA (Saraceno et al., 2010, 2012).

### Perinatal Asphyxia is Associated with a Reduced Expression of NAPE-PLD and PPARα

When we studied the expression of enzymes responsible for synthesis (DAGLα and NAPE-PLD) and degradation (FAAH) of endocannabinoids and receptors related to endocannabinoids (CB1 and PPARα), we observed a strong decrease in the expression of NAPE-PLD and PPARα in the dorsal hippocampus (**Figure 4A**, lower panel; **4B**, right panel; **5A**, lower panel and **5B**, right panel). This reduced expression could not be better explained by the cesarean section because it was specific for the asphyctic group. NAPE-PLD is the primary enzyme that catalyzes the release of AEs and consequently the basal levels of endocannabinoid AEA and other non-cannabinoid congeners, such as OEA and PEA, could be affected in the cerebral areas studied. In relation to PPARα, this receptor has been reported to mediate the neuroprotective effect of non-cannabinoid AEs in cognitive impairment, inflammation, neurodegeneration and neuronal damage after ischemia (Ahmad et al., 2012; Zhou et al., 2012; Yang et al., 2015). Taken together, the reduction of both NAPE-PLD and PPARα may indicate that AEs with no cannabinoid activity are affected in hippocampus of asphyctic animals. This finding is relevant because OEA and PEA have shown neuroprotective effects in adult cerebral ischemia (Ahmad et al., 2012; Zhou et al., 2012). Interestingly, we have recently reported a co-expression of NAPE-PLD and PPARα in hippocampal subpopulations of neurons in the rat and this may implicate a coordinated regulation of the biological roles of the PPARα signaling system through the release of OEA/PEA (Rivera et al., 2014b). Moreover, the decrease in PPARα and NAPE-PLD levels may be linked to the increment in the number of astrocytes since it has been previously shown that cannabinoids as well as PEA, and its nuclear receptor PPARα, are implicated in the inhibition of astrocytic activation in the hippocampus (Xu et al., 2006; Esposito et al., 2007; Scuderi et al., 2011, 2012). This decrease in PPARα is in accordance with previous results in which hypoxia inducible factor 1-alpha (HIF-1α) has been proven to interact with PPARα during a hypoxic event down regulating the expression of the latter (Narravula and Colgan, 2001). Moreover, the decrease in NAPE-PLD levels is also consistent with previous data that have shown a reduced activity of the enzyme after cerebral ischemia despite presenting no changes in mRNA quantities after the ischemic insult (Degn et al., 2007). According to our results this decrease in NAPE-PLD activity could be due to changes in post-transcriptional regulation of the protein resulting in diminished levels of said enzyme in hippocampus. Due to the fact that NAPE-PLD is necessary to synthesize AEs, such as the endocannabinoid AEA, changes in this enzyme levels could lead to modifications in different components of the ECS. In relation to this it is important to mention that we did not observe changes in CB1 receptor levels (**Figure 5A**, upper panel, **5B**, left panel). Therefore, further studies are needed to determine the mechanisms by which hypoxia dysregulates the ECS/AEs signaling system in the hippocampus.

Fatty acid amide hydrolase was found to be significantly increased in the C+ group in comparison with CTL and PA groups (**Figures 6A,B**). But in contrast to NAPE-PLD and PPARα, the increment of FAAH was restricted to the CA3 area of the hippocampus. This precludes us to draw a conclusion about their potential role in cesarean delivery.

### Reduced Expression of NAPE-PLD and PPARα May be Involved in PA-induced Hippocampal Neurogenesis

It is well-known that after a perinatal or adult hypoxia-ischemia episode an increment of hippocampal neurogenesis takes place (Türeyen et al., 2002; Morales et al., 2008). Although mechanisms underlying this neurogenic response are poorly understood, it is believed that its function is to try to repair the damage produced by the reduction of oxygen supply and/or reoxygenation. In the model of perinatal hypoxia-ischemia employed in the present study, hippocampal neurogenesis reaches its peak 1 week after PA which coincides with the seventh postnatal day since PA is produced at delivery (Morales et al., 2008). At the 30th postnatal day, cell proliferation is still increased (Morales et al., 2008). Since asphyctic rats showed memory impairments 1 month after PA, it could be concluded that the enhanced hippocampal neurogenesis did not translate into functional recovery. Another possibility is that this increased neurogenic response could be an aberrant phenomenon that may be responsible, in part, of the memory dysfunction (Shetty, 2014). Specific experiments are needed to know which of these two scenarios are more likely to underlie memory impairment after PA. On the other hand, ECS/AEs are involved in the control of neurogenesis (Rivera et al., 2015). Therefore, it is possible that some of the alterations found in the ECS/AEs in the present study could mediate the increment of hippocampal neurogenesis reported in this model of PA. Rivera et al. (2015) recently reported that pharmacological inhibition of FAAH, which was accompanied by increased plasma levels of OEA, PEA and AEA, was associated to a reduction of neurogenic cell proliferation in the hippocampus. The reduced hippocampal levels of NAPE-PLD and PPARα observed in the present study suggest reduced levels of AEs, such as OEA and PEA, and reduced AEA activity (by the so-called “entourage effect”), and thus could be one of the mechanisms that underlies increase hippocampal neurogenic response after PA.

## Conclusion

The most remarkable findings of the present study are that PA is associated with: (i) First, a potent reduction of the expression of the enzyme responsible of the synthesis of AEs (NAPE-PLD) in CA1 and CA3 hippocampal areas; (ii) Second, a reduction of the expression of the nuclear receptor PPARα, which mediates the action of AEs with no endocannabinoid activity, in both hippocampal areas CA1 and CA3. In addition, asphyctic rats showed impaired recognition and spatial reference memory that were accompanied by hippocampal astrogliosis without neuronal loss. Overall, these results may indicate a dysregulation of non-cannabinoid AEs in the hippocampus of postweaned asphyctic rats and encourage conducting future studies using AEs as potential neuroprotective compounds in animal models of PA.

## Author Contributions

EB, PG, FC, and FRF conceived and designed the study; EB, PG, MH, JR, TL, PR, and JS acquired and analyzed the data; EB, PG, FP, JS, FC, and FRF interpreted the data; EB, PG, MH, FP, FC, and FRF wrote the manuscript; all listed authors critically revised the manuscript and made substantial contributions to the intellectual content of the paper; all authors approved the final version of the manuscript and agreed to be accountable for all aspects related to the work in ensuring that questions about the accuracy or integrity of any part of the work are appropriately investigated and resolved.

## Conflict of Interest Statement

The authors declare that the research was conducted in the absence of any commercial or financial relationships that could be construed as a potential conflict of interest.
