# Supplementary material for: Perinatal asphyxia results in altered expression of the hippocampal acylethanolamide/endocannabinoid signaling system associated to memory impairments in postweaned rats
Source: Front Neuroanat. 2015 Nov 3;9:141. doi: 10.3389/fnana.2015.00141 (PMC4630311; doi:10.3389/fnana.2015.00141)
Supplement: Supplementary file 1 [file Data_Sheet_1.PDF]

DONKEY ANTI-RABBIT

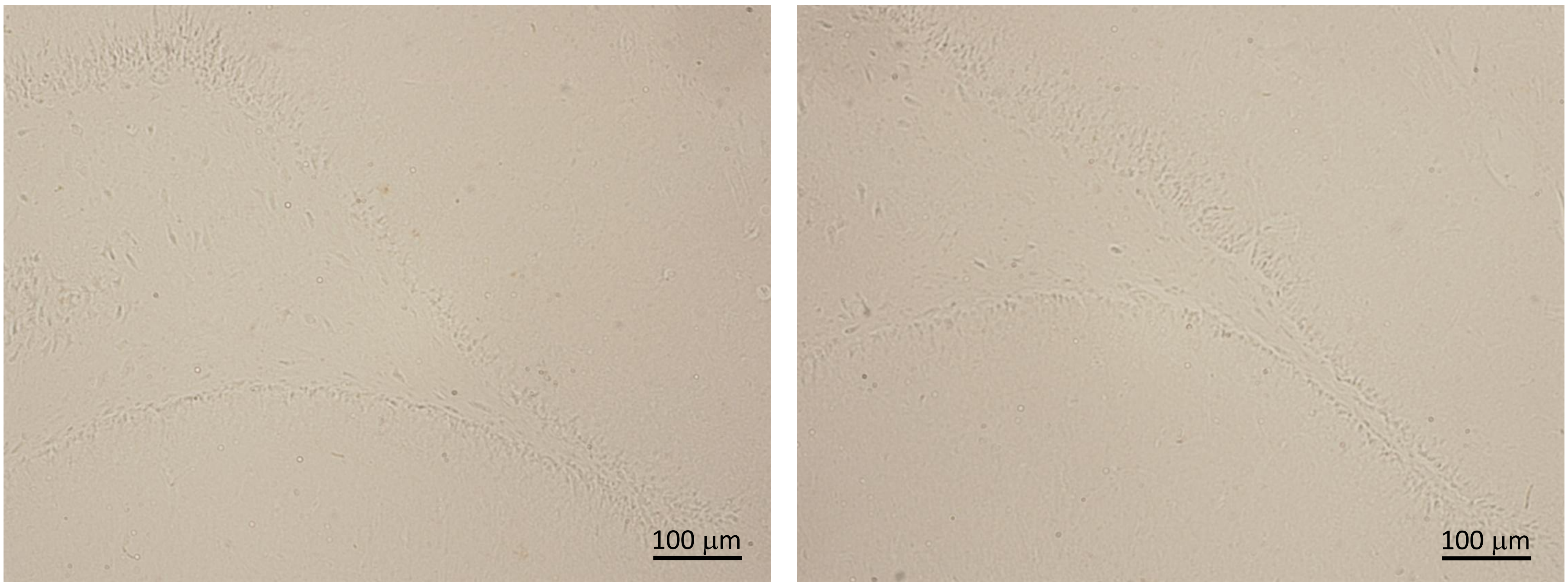

GOAT ANTI-GUINEA PIG

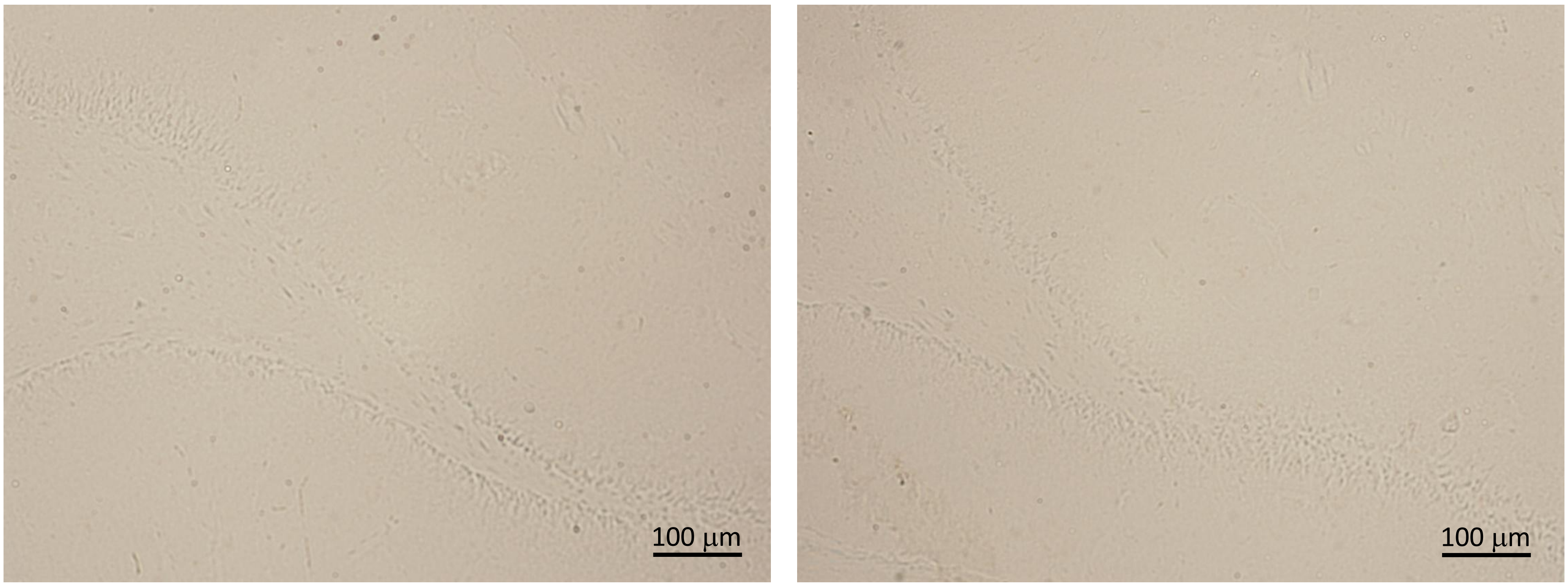

GOAT ANTI-MOUSE

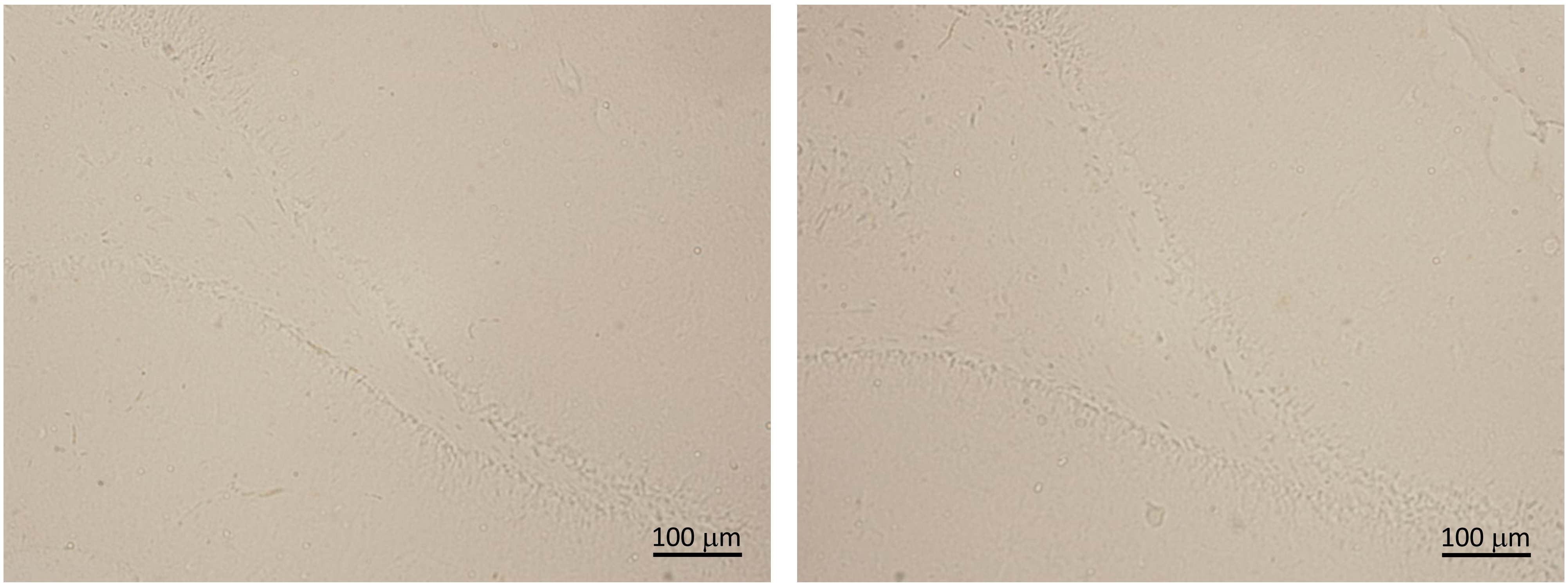

Digital high-resolution microphotographs of the dentate gyrus from control (right) and asphyxiated (left) 30-day-old rats. In all cases primary antibodies were omitted and 30 µm free floating sections were incubated with the biotinylated secondary antibody donkey anti-rabbit IgG, 5356499 (GE Healthcare) or goat anti-guinea pig, W0726 (Vector Laboratories) or goat anti-mouse IgG, 125K6063 (Sigma) in a 1:500 dilution. Background staining was not detected.
